# Laboratory-Confirmed Influenza Hospitalizations During Pregnancy or the Early Postpartum Period — Suzhou City, Jiangsu Province, China, 2018–2023

**DOI:** 10.15585/mmwr.mm7343a1

**Published:** 2024-10-31

**Authors:** Jinghui Sun, Yuanyuan Zhang, Suizan Zhou, Ying Song, Suping Zhang, Jie Zhu, Zhiyuan Zhu, Rui Wang, Hong Chen, Liling Chen, Haibing Yang, Jun Zhang, Eduardo Azziz-Baumgartner, W. William Schluter

**Affiliations:** ^1^School of Public Health, Nanjing Medical University, Nanjing, Jiangsu Province, China; ^2^Suzhou Center for Disease Control and Prevention, Suzhou City, Jiangsu Province, China; ^3^Influenza Division, National Center for Immunization and Respiratory Diseases, CDC; ^4^Suzhou Health and Family Planning Statistics Information Center, Suzhou City, Jiangsu Province, China; ^5^School of Public Health, Xuzhou Medical University, Xuzhou, Jiangsu Province, China; ^6^Chinese Center for Disease Control and Prevention, Beijing, China; ^7^Suzhou No.5 People’s Hospital, Suzhou City, Jiangsu Province, China.

**Summary**

**What is already known about this topic?**

Pregnancy is associated with increased risk for severe illness and complications attributable to influenza infection. Information about the incidence of influenza hospitalization among pregnant and early postpartum women in China is limited.

**What is added by this report?**

Population-based data from a large city in southern China estimated the annual influenza hospitalization rate to be 2.1 per 1,000 live births. Among hospitalized pregnant and postpartum women with influenza, 86% were admitted to obstetrics rather than respiratory medicine wards; fewer than one third received antiviral treatment. Influenza vaccination coverage among hospitalized pregnant and postpartum women with influenza was <0.1%.

**What are the implications for public health practice?**

Increasing vaccination coverage among pregnant women can reduce influenza-associated morbidity. Raising awareness about early detection, treatment, and infection control of influenza in obstetrics wards is needed to reduce the adverse impact of influenza on pregnant women.

## Abstract

Pregnancy is associated with increased risk for severe illness and complications associated with influenza infection. Insufficient knowledge about the risk for influenza among pregnant women and their health care providers in China is an important barrier to increasing influenza vaccination coverage and treating influenza and its complications among pregnant women. Improved influenza incidence estimates might promote wider vaccine acceptance and higher vaccination coverage. In Suzhou, active population-based surveillance during October 2018–September 2023 estimated that the annual rate of hospitalization for acute respiratory or febrile illness (ARFI) among women who were pregnant or <2 weeks postpartum was 11.1 per 1,000 live births; the annual rate of laboratory-confirmed influenza-associated ARFI (influenza ARFI) hospitalization in this group was 2.1 per 1,000 live births. A majority of hospitalized pregnant or early postpartum patients with ARFI (82.6%; 2,588 of 3,133) or influenza ARFI (85.5%; 423 of 495) were admitted to obstetrics wards rather than respiratory medicine wards. Only one (0.03%) pregnant or postpartum ARFI patient had received influenza vaccination, and 31.3% of pregnant or postpartum women hospitalized for influenza ARFI received antiviral treatment; the lowest percentage of hospitalized women with influenza ARFI who received antiviral treatment was among women admitted to obstetrics and gynecology wards (29.6% and 23.1%, respectively), compared with 54.1% of those admitted to a respiratory medicine ward. These findings highlight the risk for influenza and its associated complications among pregnant and postpartum women, the low rates of influenza vaccination among pregnant women, and of antiviral treatment of women with ARFI admitted to obstetrics and gynecology wards. Increasing awareness of the prevalence of influenza ARFI among pregnant women, the use of empiric antiviral treatment for ARFI, and the infection control in obstetrics wards during influenza seasons might help reduce influenza-associated morbidity among pregnant and postpartum women.

## Introduction

Worldwide, approximately 200 million women become pregnant each year.* Among women of reproductive age who acquire influenza, those who are pregnant are most likely to experience severe influenza-associated illness (*1*). Despite recommendations by public health agencies, including those in China (*2*), that pregnant women receive an influenza vaccine, in multiple countries where these vaccines are manufactured, licensed, and widely available, influenza vaccination coverage in this population is typically low (*3*,*4*). Insufficient information about the risk for influenza among pregnant women might contribute to reduced demand for vaccines, and passive sentinel surveillance often underestimates risk because of lack of clarity about catchment areas, insufficient testing, and underreporting (*5*). To estimate the risk for influenza illness in pregnant and postpartum women and to document the proportion of these women who were vaccinated against influenza or received antiviral medications during hospitalization for influenza, analysis of population-based surveillance of influenza hospitalizations among pregnant and early postpartum women was conducted in Suzhou, China.

## Methods

### Data Source

The data in this analysis were derived from active population-based surveillance of influenza-associated hospitalizations conducted in Suzhou (population approximately 13 million), a prefecture-level city in China’s southern Jiangsu Province, during October 2018–September 2023. The population under surveillance included all pregnant women who sought care in Suzhou and who also were found in the medical record information system, which includes all medical institutions in Suzhou. Cases of acute respiratory or febrile illness (ARFI) among female patients of reproductive age were identified using *International Classification of Diseases, Tenth Revision* codes.^†^ Inclusion criteria also included documentation of body temperature ≥99.1°F (≥37.3°C) at the time of admission. A wide range of codes and a low temperature threshold were used to capture as many illnesses as possible that were compatible with influenza infection. Pregnancy status was recorded, and nasopharyngeal swabs were collected from all pregnant women and those with a live birth within the preceding 2 weeks (early postpartum) who were hospitalized with ARFI. Laboratory-confirmed influenza-associated ARFI (influenza ARFI) was defined as ARFI with influenza RNA detected by reverse transcription–polymerase chain reaction (RT-PCR) testing of a nasopharyngeal swab.^§^ Data on live births were obtained from the Suzhou Bureau of Statistics.^¶^

### Data Analysis 

The annual ARFI hospitalization rate (ARFI hospitalizations per 1,000 live births) was calculated as the annual number of pregnant or postpartum women hospitalized with ARFI divided by the annual number of live births and multiplied by 1,000. Similarly, the annual influenza ARFI hospitalization rate (influenza ARFI hospitalizations per 1,000 live births) was calculated as the annual number of pregnant or postpartum women hospitalized with influenza ARFI divided by the annual number of live births and multiplied by 1,000. To estimate the total ARFI and influenza ARFI rates (with 95% CIs) among pregnant women in Suzhou, the ratio and 95% CI of influenza hospitalizations to total influenza illnesses (i.e., those that were and were not medically attended) from a 2022 cohort study (*3*) in Suzhou (3.2%; 95% CI = 1.5%–4.9%) was applied, using bootstrapping. This study was reviewed and approved by the Institutional Review Board of the Chinese Center for Disease Control and Prevention.

## Results

### Participants and Laboratory Testing

A total of 3,329 pregnant and postpartum women in Suzhou were hospitalized with ARFI** during the analysis period, 3,133 (94.1%) of whom had a nasopharyngeal specimen collected (Table 1). Among those who received testing, 495 (15.8%) received a diagnosis of influenza ARFI. Nearly two thirds of patients (325; 65.7%) were infected with an influenza A virus, including 163 (32.9%) with subtype A(H3N2) and 162 (32.7%) with subtype A(H1N1)pdm09. Approximately one third (167; 33.7%) of patients were infected with an influenza B virus, with Victoria lineage virus infection accounting for 157 (94.0% of all influenza B cases and 31.7% of all influenza ARFI cases among pregnant and postpartum women). Among the pregnant and postpartum influenza ARFI patients, 53 (10.7%) cases occurred during the first trimester of pregnancy, 40 (8.1%) during the second trimester, 392 (79.2%) during the third trimester, and 10 (2.0%) during the early postpartum period.

**TABLE 1:** Influenza acute respiratory or febrile illness hospitalization rate and dominant influenza viruses among pregnant or postpartum women* — Suzhou, China, 2018–2023

Metric	Analysis period					
	Oct 2018–Sep 2019	Oct 2019–Sep 2020	Oct 2020–Sep 2021	Oct 2021–Sep 2022	Oct 2022–Sep 2023	Overall
No. of ARFI hospitalizations	965	570	431	464	899	**3,329**
No. of live births	68,487	61,916	66,068	53,296	49,724	**299,491**
Annual ARFI hospitalizations per 1,000 live births (95% CI)	14.1 (13.2–15.0)	9.2 (8.5–10.0)	6.5 (5.9–7.2)	8.7 (7.9–9.5)	18.1 (16.9–19.3)	**11.1 (10.7–11.5)**
No. of sampled and tested ARFI hospitalizations (%)	878 (91.0)	526 (92.3)	417 (96.8)	452 (97.4)	860 (95.7)	**3,133 (94.1)**
No. of influenza ARFI hospitalizations (%)	233 (26.5)	99 (18.8)	3 (0.7)	77 (17.0)	83 (9.7)	**495 (15.8)**
Annual influenza ARFI hospitalizations per 1,000 live births (95% CI)	3.4 (3.0–3.9)	1.6 (1.3–2.0)	0.05 (0.01–0.13)	1.4 (1.1–1.8)	1.7 (1.3–2.1)	**2.1 (1.9–2.3)^†^**
Estimated total annual ARFI cases per 1,000 live births (95% CI)^§,¶^	440.3 (303.7–933.9)	287.8 (196.7–616.9)	203.8 (141.1–440.3)	272.2 (188.7–589.4)	565.0 (391.4–1,204.7)	**347.5 (240.3–731.5)**
Estimated total annual influenza ARFI cases per 1,000 live births (95% CI)^§,¶^	106.3 (72.6–236.3)	50.0 (33.4–116.4)	1.6 (0.5–7.2)	45.0 (29.4–107.8)	52.2 (34.3–123.3)	**65.9 (45.2–142.4)^†^**
Dominant influenza viruses**	A(H1N1)pdm09	B/Victoria	B/Victoria	A(H3N2) and B/Victoria	A(H1N1)pdm09 and A(H3N2)	**A(H1N1)pdm09, A(H3N2), and B/Victoria**

**Abbreviations:** ARFI = acute respiratory or febrile illness; influenza ARFI = laboratory-confirmed influenza-associated acute respiratory or febrile illness.

* <2 weeks postpartum.

^†^ Because influenza activity during 2020–21 did not achieve epidemic levels, these data were excluded from the calculation of the average. The start of each influenza epidemic period was defined as the first day of 3 consecutive influenza reporting weeks in which the percentage of specimens testing positive for any influenza virus infection exceeded 5%. The end of each influenza epidemic period was defined as the day before the first of 3 consecutive influenza reporting weeks during which the percentage of specimens testing positive for influenza was <5%.

^§^ The total annual ARFI or influenza ARFI rates were estimated through observed hospitalization rates divided by the percentage of hospitalizations among the total number of pregnant or postpartum women with ARFI or influenza ARFI. The percentages of hospitalizations among the total number of pregnant or postpartum women with ARFI or influenza ARFI were assumed the same and equal to the percentage of hospitalizations among the total number of pregnant or postpartum women with influenza (3.2%; 95% CI = 1.5%–4.9%). https://pubmed.ncbi.nlm.nih.gov/34323381

^¶^ Including cases that were and were not medically attended, outpatients, and inpatients.

** Dominant influenza viruses were defined as those 1) accounting for ≥70% of all isolates during the season or 2) accounting for 40%–70% of all isolates, and the second most common virus accounted for <30%. Subtype/lineage was considered as codominant with the most common virus if it accounted for ≥30% of all isolates.

### Hospitalization Rates

Among the 495 hospitalized pregnant or postpartum women with influenza ARFI, 479 (96.8%) cases occurred during periods when influenza detection exceeded the epidemic threshold (Figure). Influenza ARFI hospitalization rates among pregnant and postpartum women were highest during 2018–2019 (3.4 per 1,000 live births), and lowest during 2020–2021 (0.05 per 1,000 live births). Reported influenza cases in this group of women during 2020–2021, following implementation of COVID-19 nonpharmaceutical interventions (NPIs),^††^ were markedly lower than that during other years (p<0.05) and never reached the epidemic threshold. When influenza ARFI hospitalizations among pregnant or postpartum women during 2020–2021 were excluded from the analysis, the average maternal influenza ARFI hospitalization rate for the remaining four influenza seasons was 2.1 per 1,000 live births. The annual average ARFI hospitalization rate was 11.1 per 1,000 live births, including during 2020–2021 (Table 1).

**FIGURE:**
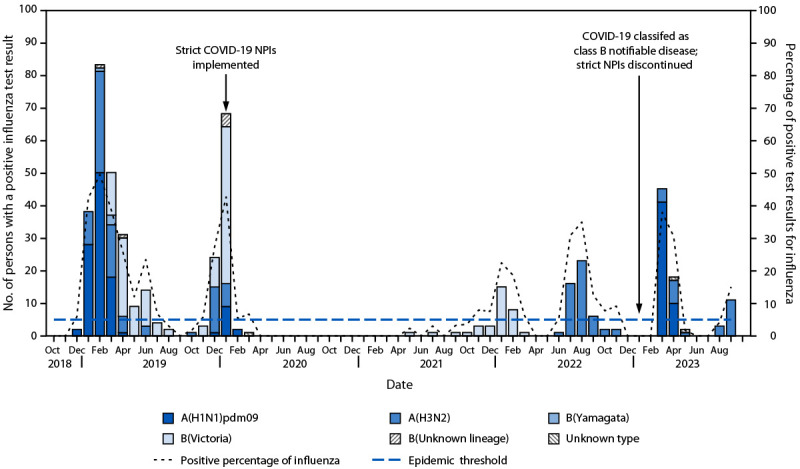
Dates of hospitalization of pregnant or postpartum* women, distribution of identified influenza virus subtypes, and implementation of COVID-19 control measures — Suzhou, China, 2018–2023^†^ **Abbreviation:** NPI = nonpharmaceutical intervention. * Less than 2 weeks postpartum. ^†^ Three classes (class A, class B, and class C) of notifiable infectious diseases in 41 categories are listed in China. Plague and cholera are listed as class A infectious diseases. Severe acute respiratory syndrome, AIDS, and tuberculosis are among the class B infectious diseases. Class C infectious diseases include influenza and mumps. https://doi.org/10.1016/j.ijid.2016.04.010

### Characteristics of Hospitalized Pregnant or Postpartum Influenza ARFI Patients

Among the 3,329 pregnant or postpartum women who were hospitalized with ARFI, including 495 (14.9%) with influenza ARFI, information on the type of hospital facility was available for 3,133 (94.1%) with ARFI, including all 495 with influenza ARFI (15.8% of the 3,133 with available information). Overall, 2,680 (85.5%) ARFI patients and 423 (85.5%) influenza ARFI patients were admitted to grade III medical institutions, the highest acuity treatment level, which typically treat the most severe cases of illness in China’s three-tier health care system^§§^ (Table 2). A majority of pregnant and postpartum women with ARFI or influenza ARFI were admitted to obstetrics wards (2,588; 82.6% and 423; 85.5%, respectively), rather than to a respiratory medicine ward (299; 9.5% and 37; 7.5%, respectively). Among influenza ARFI patients admitted to obstetrics wards, 371 (87.7%) were in their third trimester.

**TABLE 2:** Distribution of hospital facilities and wards that cared for pregnant or postpartum* patients with acute respiratory or febrile illness and receipt of antiviral treatment, by hospital grade and ward — Suzhou, China, 2018–2023

Hospital ward	Institution grade,^†^ no. (%)^§^							
	Total		Grade I		Grade II		Grade III	
	Total admitted	Received antiviral treatment	Total admitted	Received antiviral treatment	Total admitted	Received antiviral treatment	Total admitted	Received antiviral treatment
**ARFI patients**								
**Total**	**3,133 (100)** ^¶^	**331 (10.6)**	**24 (100)**	**0 (—)**	**429 (100)**	**25 (5.8)**	**2,680 (100)**	**306 (11.4)**
Obstetrics	**2,588 (82.6)**	**258 (10.0)**	**13 (54.2)**	0 (—)	**348 (81.1)**	20 (5.7)	**2,227 (83.1)**	238 (10.7)
Respiratory medicine	**299 (9.5)**	**57 (19.1)**	**1 (4.2)**	0 (—)	**23 (5.4)**	4 (17.4)	**275 (10.3)**	53 (19.3)
Gynecology	**173 (5.5)**	**7 (4.0)**	**10 (41.7)**	0 (—)	**54 (12.6)**	1 (1.9)	**109 (4.1)**	6 (5.5)
Others	**73 (2.3)**	**9 (12.3)**	**0 (—)**	0 (—)	**4 (0.9)**	0 (—)	**69 (2.6)**	9 (13.0)
**Influenza ARFI patients**								
**Total**	495 (100)**	**155 (31.3)**	**1 (100)**	**0 (—)**	**71 (100)**	**14 (19.7)**	**423 (100)**	**141 (33.3)**
Obstetrics	**423 (85.5)**	**125 (29.6)**	**0 (—)**	0 (—)	**62 (87.3)**	10 (16.1)	**361 (85.3)**	115 (31.9)
Respiratory medicine	**37 (7.5)**	**20 (54.1)**	**0 (—)**	0 (—)	**4 (5.6)**	3 (75.0)	**33 (7.8)**	17 (51.5)
Gynecology	**26 (5.3)**	**6 (23.1)**	**1 (100)**	0 (—)	**5 (7.0)**	1 (20.0)	**20 (4.7)**	5 (25.0)
Others	**9 (1.8)**	**4 (44.4)**	**0 (—)**	0 (—)	**0 (—)**	0 (—)	**9 (2.1)**	4 (44.4)

**Abbreviations:** ARFI = acute respiratory or febrile illness; influenza ARFI = laboratory-confirmed influenza-associated acute respiratory or febrile illness.

* <2 weeks postpartum.

^†^ Hospitals in China are classified into three grades. The lowest is the grade I hospital, which includes primary hospitals and health centers that directly provide prevention, medical care, and rehabilitation services to communities with a certain population. A grade II hospital is a regional hospital that provides comprehensive medical and health services to multiple communities and undertakes certain teaching and research tasks. The highest rank is the grade III hospital, which is a large-scale general hospital with more than 500 beds that integrates medical service, education, and research functions.

**^§^** Percentages in Total admitted columns are column percentages; percentages in Received antiviral treatment columns are percentages of the total number of patients hospitalized in each grade and ward type (e.g., among 348 patients hospitalized in grade II obstetrics wards, 20 (5.7%) received antiviral treatment).

^¶^ Number of patients (i.e., 94% of 3,329) who had a nasopharyngeal swab collected and for whom information on hospitalization ward was available.

** The 495 influenza ARFI patients were a 15.8% subset of the 3,133 ARFI patients.

Among all 3,329 pregnant women with ARFI, only one (0.03%) had received an influenza vaccination, and this vaccinated patient received a negative test result for influenza. Fewer than one third of pregnant or postpartum patients with influenza ARFI (155; 31.3%) received influenza antiviral drug treatment before or during hospitalization. Among 423 women with influenza ARFI hospitalized in an obstetrics ward, 125 (29.6%) received antiviral drug treatment; the highest percentage of women with influenza ARFI admitted to an obstetrics ward who received antiviral treatment were those admitted to a grade III facility (115 of 361 [31.9%]). Among 37 pregnant or postpartum women with influenza ARFI admitted to a respiratory medicine ward, 20 (54.1%) received antiviral treatment. Among 495 influenza ARFI patients, nine (1.8%) were admitted to an intensive care unit; no mechanical ventilation or death cases were reported during hospitalization.

### Estimated Total Influenza ARFI Incidence

Estimated total annual influenza ARFI incidence among pregnant and postpartum women, including inpatients or outpatient cases that were and were not medically attended was 65.9 per 1,000 live births. Total annual ARFI incidence was 347.5 cases per 1,000 live births (Table 1).

## Discussion

Analysis of active population-based surveillance data for hospitalized pregnant or early postpartum women in Suzhou, China found an annual influenza ARFI hospitalization rate of 2.1 per 1,000 live births. A majority of patients were admitted to obstetrics hospital wards, which are not typically included in respiratory disease surveillance. Influenza ARFI incidence among pregnant and postpartum women, including cases that were and were not medically attended, was estimated to be approximately 70 per 1,000 live births. Only one pregnant woman hospitalized with ARFI had documentation of receipt of influenza vaccination. Fewer than one third of those hospitalized for influenza ARFI were treated with antiviral medications; among patients admitted to obstetrics and gynecology wards, fewer than one third received antiviral medications, compared with approximately one half of those who were admitted to respiratory medicine wards.

The annual influenza ARFI hospitalization rate provided important information about influenza-associated inpatient care needs of pregnant or early postpartum women in China. The sentinel influenza surveillance systems in China, which mainly target respiratory medicine wards, have no data collected on pregnancy status or no defined catchment population, given that a majority of sentinel hospitals are large referral hospitals. The clinical diagnosis–based nationally notifiable disease reporting system in China does not report pregnancy status and is subject to significant undertesting and underreporting (*6*). Comparison of influenza hospitalization rates across countries is challenging because of differences in health care–seeking behavior and health care systems. However, the annual rate of community influenza ARFI is more easily compared. The total annual rate of community influenza ARFI among pregnant and early postpartum women (65.9 per 1,000 live births), estimated using data from a 2022 cohort study in Suzhou (*3*), was equivalent to approximately 0.7 cases per 100 person-months (6.6 per 100 person-years). This rate is comparable to recent estimates from community-based prospective cohorts from El Salvador and Panama (5.0 per 100 person-years) (*7*), Kenyan cohorts (0.9–1.2 per 100 person-months) (*8*), the China respiratory illness surveillance among pregnant women cohort (0.7–2.1 per 100 person-months) (*3*), and pregnancy and influenza multinational epidemiologic cohorts from India, Peru, and Thailand (0.7–0.9 per 100 person-months) (*9*), with slight differences possibly attributed to seasonal and geographic variations in this population.

A majority of hospitalized pregnant and postpartum women with ARFI or influenza ARFI were admitted to obstetrics wards, highlighting the importance of including maternity and postnatal wards and departments in sentinel surveillance to estimate influenza ARFI incidence. These estimates suggest that relying on traditional respiratory medicine ward surveillance would have missed approximately 85% of influenza hospitalizations among pregnant or postpartum patients. A strength of this evaluation is that it covered all hospitals in Suzhou and provided testing for influenza by RT-PCR for all pregnant or postpartum patients with ARFI. The methods described here could be used in other settings to accurately estimate the morbidity associated with severe influenza among pregnant women. Accurate estimates can help guide vaccination efforts in groups at risk for severe illness, as well as treatment of pregnant and postpartum women with influenza.

Based on influenza vaccine effectiveness data (*10*), approximately 40% of maternal influenza hospitalizations would have been vaccine-preventable; however, pregnant women were rarely vaccinated. Influenza vaccination coverage among the approximately 18 million pregnant women in China each year^¶¶^ is 0.04% (95% CI = 0.02%–0.08%), in part because awareness about the risk for influenza illness is low coupled with a lack of demand for influenza vaccination (*3**,**5*). This study confirmed that influenza vaccination coverage among hospitalized pregnant or postpartum women in Suzhou is low. Educating obstetricians about the risks associated with influenza in pregnancy and encouraging them to provide a strong influenza vaccination recommendation for women who are or will be pregnant during the influenza season could help prevent severe influenza morbidity.

The study further found that fewer than one third of pregnant or postpartum patients in Suzhou were treated with influenza antiviral medication even after the diagnosis of influenza; these percentages were lowest among pregnant and postpartum women hospitalized in obstetrics and gynecology wards. Cost and limited availability of the medications, as well as concerns about potential side effects of treatment or risk to the fetus might also have contributed to low antiviral drug treatment for influenza in this population, although multiple observational studies of treatment with oral oseltamivir or zanamivir during pregnancy have not shown a risk to the fetus.*** In addition to addressing safety concerns, cost-benefit evaluation of antiviral drug treatment for persons at increased risk for influenza complications who seek care at, for example, urgent care centers, might also help increase use of antiviral drugs and limit the occurrence of severe illness. The study findings warrant educating health care providers, especially those working in obstetrics wards, about treatment with antivirals for pregnant or postpartum women with influenza.

These estimates could also be incorporated into vaccine and antiviral cost-benefit analyses to help health authorities assess the return on investment, particularly when compared with more familiar traditional Chinese medicines and supportive care, and to assess the costs and benefits of NPIs (e.g., maintaining good respiratory hygiene, avoiding close contact with persons who have signs or symptoms of influenza-like illness, and minimizing gatherings in crowded places). Implementation of such NPIs during the COVID-19 pandemic might have reduced the risk for infection and spread of influenza.

### Limitations

The findings in this report are subject to at least two limitations. First, the study was conducted in a single large city, and the findings might not be generalizable to the rest of China. The study site is economically developed, and health-seeking behavior might vary in other parts of the country. However, it is expected that other, less developed areas with insufficient supplies of vaccine, testing kits, or antiviral drugs, would also experience substantial influenza illnesses and would be less likely to use these specific tools for early detection and protection of pregnant women against influenza. Therefore, these findings might underestimate the incidence of influenza ARFI among pregnant and postpartum women in other parts of the country. Second, the study design did not allow for the inclusion of patients with influenza who might have died or had a fetal loss associated with the hospitalization, which might have underestimated the severe impact of failure to vaccinate and treat pregnant women with influenza.

### Implications for Public Health Practice

The population-based active surveillance outlined in this report underscores the substantial risk for influenza illness among pregnant and postpartum women in China and the potential benefit to pregnant women of offering annual influenza vaccination in prenatal care facilities. Influenza in pregnant women is associated with higher morbidity and mortality. In addition, pregnant women with influenza-like illness might not seek care in respiratory clinics or wards. Increasing awareness of when to seek care for suspected influenza illness, the benefits of early detection and treatment, and infection control in facilities, including prenatal care clinics or wards, could help reduce maternal morbidity during influenza epidemics. Receipt of annual influenza vaccination by pregnant women can prevent influenza-associated morbidity and hospitalization (*10*).

## Footnotes

*
https://population.un.org/wpp

† The *International Classification of Diseases, Tenth Revision* codes to identify ARFI included the following: A41 (sepsis, unspecified), B34 (viral infection, unspecified), B95.3 (*Streptococcus pneumoniae*), B96.0 (*Mycoplasma pneumoniae*), B96.1 (*Klebsiella pneumoniae*), B96.3 (*Haemophilus influenzae*), B97.0 (Adenovirus), B97.2 (Coronavirus), B97.3 (Retrovirus), B97.4 (respiratory syncytial virus), B97.8 (other viral agents), B99.x01 (other unspecified infectious diseases), J00–J06 (acute upper respiratory infectious), J09–J18 (influenza and pneumonia), J20–J22 (other acute lower respiratory infections), J35 (chronic diseases of the tonsils and adenoids), J36 (peritonsillar abscess), J39 (other diseases of the upper respiratory tract), J40 (bronchitis, not acute or chronic), J45 (asthma), J46 (status asthmaticus), J80 (acute respiratory distress syndrome), J81 (pulmonary edema), J84 (pulmonary fibrosis, unspecified), J86.9 (pyothorax without fistula), J90 (pleural effusion), J96 (respiratory failure, not classified elsewhere), J98 (other respiratory disorders), O75.1 (shock during or after labor and delivery), O75.2 (pyrexia during labor, not elsewhere classified), O86.4 (pyrexia of unknown origin following delivery), O98.5 (other viral diseases complicating pregnancy, childbirth, and the puerperium), O98.8 (other maternal infectious and parasitic diseases complicating pregnancy, childbirth, and the puerperium), O99.5 (diseases of the respiratory system complicating pregnancy, childbirth, and the puerperium), R04–R07 (hemorrhage from respiratory passages, cough, abnormalities of breathing, pain in throat and chest), R09 (other symptoms and signs involving the circulatory and respiratory system), R50 (fevers of unknown or other origins), R57.9 (shock, unspecified), R65 (systemic inflammatory response syndrome), and R68.8 (other general symptoms and signs).

§
https://ivdc.chinacdc.cn/cnic/fascc/201802/P020180202290930853917.pdf

¶
https://tjj.suzhou.gov.cn

** Excluding those seen only in emergency departments but not admitted.

†† NPIs to contain COVID-19 in China included three major groups: 1) the restriction of intercity population movement; 2) the identification and isolation of cases, contact tracing, and quarantine of exposed persons; and 3) the reduction of inner-city travel and contact to increase social distance (e.g., school and workplace closures and cancellation of mass gatherings). https://www.nature.com/articles/s41586-020-2293-x

§§
https://www.semanticscholar.org/paper/The-Different-Classification-of-Hospitals-Impact-on-Li-Du/937ebf09b1ee8dfbfc18e57ca429d3db8f018325

¶¶
https://www.unfpa.org/data/sowmy/CN

***
https://www.cdc.gov/flu/professionals/antivirals/avrec_ob.htm
